# Morbillivirus and Pilot Whale Deaths, Canary Islands, Spain, 2015

**DOI:** 10.3201/eid2204.150954

**Published:** 2016-04

**Authors:** Eva Sierra, Antonio Fernández, Cristian Suárez-Santana, Aina Xuriach, Daniele Zucca, Yara Bernaldo de Quirós, Natalia García-Álvarez, Jesús De la Fuente, Simona Sacchini, Marisa Andrada, Josué Díaz-Delgado, Manuel Arbelo

**Affiliations:** University of Las Palmas de Gran Canaria, Arucas (Las Palmas), Canary Islands, Spain

**Keywords:** morbillivirus, viruses, pilot whales, cetaceans, cetacean morbillivirus, pilot whale morbillivirus, PWMV, deaths, Canary Islands, Spain

**To the Editor:** Four strains of cetacean morbillivirus (CeMV; family *Paramyxoviridae*, genus *Morbillivirus*) have been detected in the global cetacean population: porpoise morbillivirus (*1*), dolphin morbillivirus (*2*), pilot whale morbillivirus (PWMV) (*3*), and Longman’s beaked whale morbillivirus (*4*). In addition, 2 novel CeMV sequences or strains isolated from the Indo-Pacific bottlenose dolphin (*Tursiops aduncus*) and the Guiana dolphin (*Sotalia guianensis*) have been recently reported in the Southern Hemisphere (*5**,**6*).

Pilot whales are known to be susceptible to 2 strains of CeMV, PWMV, and dolphin morbillivirus (*3**,**7**,**8*). Only 2 deaths of whales have been reported to be caused by PWMV: 1 long-finned pilot whale (*Globicephala melas*) (*3*) and 1 short-finned pilot whale (*G. macrorhynchus*) (*8*). We report deaths of 3 short-finned pilot whales caused by PWMV in the northeastern Atlantic Ocean along the coast of the Canary Islands, Spain.

During mid-January–May 2015, a total of 3 whales (animals 1, 2, and 3) were found dead along the coasts of the Canary Islands (Table). Complete standardized necropsy was performed for all whales. Tissue samples from animals 1 and 2 were fixed in 10% neutral-buffered formalin for histologic and immunohistochemical analyses (Technical Appendix Figure). Immunohistochemical analysis was performed (brain, intestine, lymph nodes, lung, kidney, adrenal gland, uterus, ovary, testis, and spleen) by using a monoclonal antibody against the nucleoprotein of canine distemper virus (CDV-NP; VMRD, Inc., Pullman, WA, USA) (*7*). Samples of lung, pulmonary lymph nodes, larynx, laryngeal tonsil, intestine, spleen, and brain were frozen (−80°C) for virologic analysis.

**Table:** Characteristics for 3 short-finned pilot whales stranded along the Canary Islands, Spain, 2015*

Animal no., age/sex	Total body length, cm	Weight, kg	Date stranded	Location	Decomposition code	Main pathologic findings	IHC-positive result	PCR-positive result
1, J/F	202	119	Jan 14	Fuerteventura	Moderate autolysis	Dermatitis, suppurative rhinitis, paranasal sinusitis, otitis media, air sacculitis, laryngitis, laryngeal tonsillitis, BIP, hyperkeratotic gastritis, lymphoid depletion	Lung, larynx, laryngeal tonsil, lymph nodes, spleen, adrenal gland, keratinized stomach, intestine, uterus, ovary	Lung, **larynx, pulmonary lymph node, laryngeal tonsil, spleen, intestine**
2, C/M	168	75	May 15	Fuerteventura	Fresh	Dermatitis, cheilitis, suppurative laryngeal tonsillitis, BIP, encephalitis	Lung, laryngeal tonsil, lymph nodes, spleen, kidney, testis, epididymis, intestine, adrenal gland, urinary bladder, brain	Lung, pulmonary lymph node, laryngeal tonsil, intestine, brain
3, A/M	448	900	May 20	Tenerife	Advanced autolysis	Advanced autolysis	None	Laryngeal tonsil

*IHC, immunohistochemical analysis; J, juvenile; BIP, bronchointerstitial pneumonia; C, calf; A, adult. Tissues that were positive for herpesvirus and morbillivirus simultaneously are indicated in bold.

Grossly, the most remarkable findings in animal 1 were severe suppurative rhinitis, with clogged nasal passages by the accumulation of large quantity of purulent material, otitis media, sacculitis, and laryngitis. Severe diffuse epithelial hyperplasia and hyperkeratosis was observed along the upper respiratory tract and keratinized stomach. Animal 2 had severe proliferative dermatitis and cheilitis, and severe, suppurative, laryngeal tonsillitis. Animal 3 had advanced autolysis, which precluded pathologic analysis.

Histologically, moderate, multifocal, bronchointerstitial pneumonia, severe suppurative tonsillitis and systemic lymphoid depletion were identified in animals 1 and 2. Severe nonsuppurative meningoencephalitis with neuronal and glial cell degeneration and necrosis, microgliosis and syncytial cells were observed in animal 2.

Immunohistochemical analysis showed morbillivirus antigen in the bronchiolar epithelium, type 2 pneumocytes, and alveolar multinucleate cells. Syncytia from lymph nodes, laryngeal tonsil, spleen, and intestine also showed positive immunolabeling for morbillivirus. Epithelial tropism caused by the virus was suggested by identification of viral antigen in epithelia of the lung, larynx, keratinized stomach, intestine, kidney, urinary bladder, epididymis, and endometrial glands. In addition, intense immunolabeling was detected in neurons (soma, dendrites, axon hillock, and axons) and glial cells, primarily throughout the cerebral gray matter of animal 2.

Molecular detection of CeMV was performed by a using a 1-step reverse transcription PCR for a 426-bp conserved region of the phosphoprotein gene (*7*). All tested samples from animals 1 and 2 and a laryngeal tonsil sample from animal 3 showed positive PCR results. Because co-infections with herpesvirus and morbillivirus were observed during morbillivirus epizootics in seals in 1988 and dolphins in 2006–2007, we also tested the same tissue for herpesvirus by conventional nested PCR (*9*). Herpesvirus DNA was detected in all samples from animal 1 except lung, although no specific lesions compatible with this infectious agent were observed.

A pool containing all morbillivirus-positive PCR amplicons for animals 1 and 2 (GenBank accession nos. KT006289 and KT006290), a PCR amplicon for the brain sample from animal 2 (GenBank accession no. KT006291), and a PCR amplicon for the larynx from animal 3 were sequenced. A BLAST search (http://www.ncbi.nlm.nih.gov/blast/Blast.cgi) showed that amplified samples were nearly identical to reference PWMV sequences (GenBank accession nos. AF200817 [*3*] and FJ842381 [*8*]). The sequence obtained from animal 3 was too short and degenerated to be accurately classified as CeMV, although it showed high homology with PWMV and porpoise morbillivirus.

It has been proposed that pilot whales might be enzootically infected with CeMV (*10*). These whales might be responsible for maintaining and transmitting CeMV over long distances or to other odontocetes. No die-offs have been observed in these species. However, an outbreak of a lethal morbillivirus infection in long-finned pilot whales caused by a dolphin morbillivirus strain occurred in the Mediterranean Sea during the end of October 2006–April 2007 (*7*).

Results of this study support the previous hypothesis that pilot whales have a species-adapted morbillivirus but indicate that lethal infections are not as rare as previously believed (*3*). The tropism of the virus in these cases, the high number of multinucleated syncytial cells, and the severity of the lesions resemble the acute systemic symptoms observed in dolphins infected with morbillivirus (*2*). Thus, pilot whales in the northeastern Atlantic Ocean could be at risk for infection, especially in one of the main pilot whale–watching regions between La Gomera and Southern Tenerife Islands in the Canary Islands, which has >700,000 visitors each year.

**Technical Appendix.** Microscopic images of tissue samples from 2 short-finned pilot whales (*Globicephala macrorhynchus*) from the eastern Atlantic Ocean stranded along the Canary Islands, Spain, 2015.

## References

[1] Barrett T, Visser IK, Mamaev L, Goatley L, van Bressem MF, Osterhaust AD. Dolphin and porpoise morbilliviruses are genetically distinct from phocine distemper virus. Virology. 1993;193:1010–2 . 10.1006/viro.1993.1217 8460473

[2] Domingo M, Visa J, Pumarola M, Marco AJ, Ferrer L, Rabanal R, Pathologic and immunocytochemical studies of morbillivirus infection in striped dolphins (*Stenella coeruleoalba*). Vet Pathol. 1992;29:1–10 . 10.1177/030098589202900101 1557861

[3] Taubenberger JK, Tsai MM, Atkin TJ, Fanning TG, Krafft AE, Moeller RB, Molecular genetic evidence of a novel morbillivirus in a long-finned pilot whale (*Globicephalus melas*). Emerg Infect Dis. 2000;6:42–5 . 10653568 10.3201/eid0601.000107 PMC2627976

[4] West KL, Sanchez S, Rotstein D, Robertson KM, Dennison S, Levine G, A Longman’s beaked whale (*Indopacetus pacificus*) strands in Maui, Hawaii, with first case of morbillivirus in the central Pacific. Mar Mamm Sci. 2013;29:767–76.

[5] Groch KR, Colosio AC, Marcondes MC, Zucca D, Diaz-Delgado J, Niemeyer C, Novel cetacean morbillivirus in Guiana dolphin, Brazil. Emerg Infect Dis. 2014;20:511–3 . 10.3201/eid2003.131557 24565559 PMC3944878

[6] Stephens N, Duignan PJ, Wang J, Bingham J, Finn H, Bejder L. 1st, et al. Cetacean morbillivirus in coastal Indo-Pacific bottlenose dolphins, Western Australia. Emerg Infect Dis. 2014;20:666–70. 10.3201/eid2004.131714 PMC3966363 24656203

[7] Fernández A, Esperon F, Herraez P, de Los Monteros AE, Clavel C, Bernabe A, Morbillivirus and pilot whale deaths, Mediterranean Sea. Emerg Infect Dis. 2008;14:792–4 . 18439363 10.3201/eid1405.070948 PMC2600256

[8] Bellière EN, Esperon F, Fernandez A, Arbelo M, Munoz MJ, Sanchez-Vizcaino JM. Phylogenetic analysis of a new Cetacean morbillivirus from a short-finned pilot whale stranded in the Canary Islands. Res Vet Sci. 2011;90:324–8 . 10.1016/j.rvsc.2010.05.038 20576281

[9] VanDevanter DR, Warrener P, Bennett L, Schultz ER, Coulter S, Garber RL, Detection and analysis of diverse herpesviral species by consensus primer PCR. J Clin Microbiol. 1996;34:1666–71 . 8784566 10.1128/jcm.34.7.1666-1671.1996 PMC229091

[10] Duignan PJ, House C, Geraci JR, Early G, Copland HG, Walsh MT, Morbillivirus infection in two species of pilot whales (*Globicephala* sp.) from the western Atlantic. Mar Mamm Sci. 1995;11:150–62. 10.1111/j.1748-7692.1995.tb00514.x

