# Supplementary material for: Morbillivirus and Pilot Whale Deaths, Canary Islands, Spain, 2015
Source: Emerg Infect Dis. 2016 Apr;22(4):740–2. doi: 10.3201/eid2204.150954 (PMC4806956; doi:10.3201/eid2204.150954)
Supplement: Supplementary file 1 — Technical Appendix. Microscopic images of tissue samples from 2 short-finned pilot whales (Globicephala macrorhynchus) from the eastern Atlantic Ocean stranded along the Canary Islands, Spain, 2015. [file 15-0954-Techapp-s1.pdf]

Article DOI: http://dx.doi.org/10.3201/eid2204.151505

Morbillivirus and Pilot Whale Deaths, Canary Islands, Spain

Technical Appendix

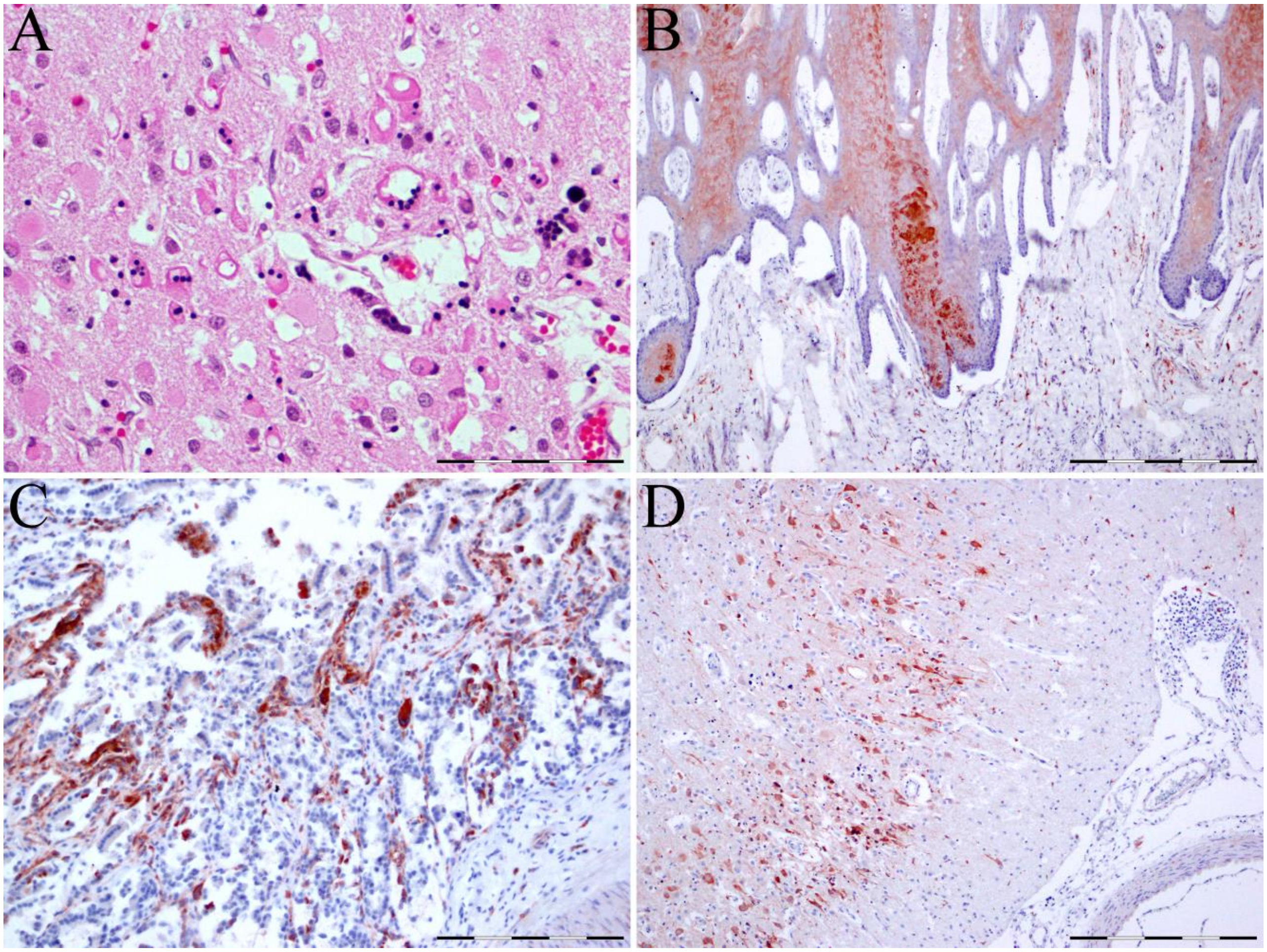

Technical Appendix Figure. Microscopic images of tissue samples from 2 short-finned pilot whales (*Globicephala macrorhynchus*) from the eastern Atlantic Ocean stranded along the Canary Islands, Spain, 2015. A) Brain of animal 2 showing degenerate neurons with cytoplasmic vacuolation, eccentric nuclei, and chromatolysis. Nuclear pyknosis is present in degenerating necrotic neurons (hematoxylin and eosin stained). Scale bar = 50 µm. B) First compartment of the stomach of animal 1 showing intense immunoperoxidase staining of morbillivirus in cytoplasm and nuclei of hyperplasic epithelial cells of the keratinized stomach. Clusters of neutrophils were present at different layers of the hyperkeratotic epithelium (avidin–biotin–peroxidase stained and Harris hematoxylin counterstained). Scale bar = 200 µm. C) Intestine of animal 1 showing intense immunoperoxidase staining of morbillivirus in cytoplasm and nuclei of epithelial and syncytial cells

(avidin–biotin–peroxidase stained and Harris hematoxylin counterstained). Scale bar = 100 μm. D) Brain of animal 2 showing intense immunostaining (neurons, glial cells, and neuronal processes) mainly confined to gray matter of cerebral cortex (avidin–biotin–peroxidase stained and Harris hematoxylin counterstained). Scale bar = 200 μm.
